# Edison’s Electric Lamp

**Published:** 1880-01

**Authors:** 


### Scientific News.

### EDISON'S ELECTRIC LAMP.

The *Cincinnati Commercial* of Dec. 21st, 1879, with characteristic energy and promptitude, published a comprehensive account of the history of Edison's experiments with electricity, for the production of a cheap, practical and reliable light. The article is illustrated with cuts, conveying to the mind of the reader an exact knowledge of the invention. Though he has met with many discouraging experiences, yet the inventor of the "phonograph, telephone, and quadruplex telegraph," has conferred upon our civilization, one of the greatest achievements of scientific research and human ingenuity, if estimated according to its adaptability to the necessities of every-day life. The subjoined extracts from the editorial columns of the *Commercial* will serve to give an idea of the lamp and its uses:

"The point of the discovery we make known this morning is, that *carbonized paper answers the purpose that it was thought*

*platinum alone could serve*, and is not only as cheap as ashes, but better than precious metal.

All difficulties but this of the composition of the wick of the electrical candle, had been overcome. The generation of electricity has become familiar. There are many well known electrical machines. The divisibility of the current has been found possible. It is as divisible under EDISON'S process, as a volume of gas.

What of the Edison Lamp? It can be made at a cost of twenty-five cents. In its simplest form it is as cheap as a gas tip. It is a plain glass globe, about the size of a small orange or large lemon. In the bottom is fitted a metallic stopper, through which the copper wires pass, and a strip of carbonized paper, shaped like a horse-shoe, connects the wires. The air is exhausted in the globe. Turn on the electricity, which is quite as simple an operation as turning on gas, and the carbon becomes and remains luminous, giving out a soft, brilliant, powerful light, and the wick endures. No limit to its endurance has been found. No matches are needed to strike a light. You touch the key and there is your illumination. The glass globe does not become heated. It grows warm, but by no possibility can there be any combustion. Break the glass, and the light is instantly and utterly extinguished. The moment the air enters, the light is out. It would be perfectly safe, therefore, to use such a light in a powder magazine. It will be of inestimable advantage on ship-board. It can be employed with the happiest results in mines heretofore extra-hazardous. It will, by at once abolishing the use of matches in our houses, and introducing a light that neither heats nor enkindles, vastly reduce the liability of fires, and thus speedily put down the rates of insurance. This light will do away with the necessity of heat in well lighted apartments. One can produce a dazzling illumination without changing the temperature."

"It is a light that does not harm the eyes, and that may be shaded and regulated at pleasure—combining the softness of the oil lamp with the splendor of the burning of carbon points.

"It is EDISON'S careful estimate that the cost of the electric light, according to this system, will be something less than one-half the cost of coal gas. He is sure it will not exceed that figure. Beyond that he proposes to use electricity as a motor, to run sewing machines and the like, and has prepared a meter for the exact measurement of the electricity furnished to each house. We need not dwell upon the importance of this discovery. It is revolutionary."

---

Demant gives the following as the results of his experiments upon a patient, in whom the operation of hermiotomy had left a fistula in the lower end of the small intestine, the canal presenting two parts, in the lower of which intestinal, juice collected. " 1st. Human intestinal juice is a thin, clear fluid, of stongly alkaline reaction. 2nd. The amount secreted is not large. During the act of digestion, more is secreted than at other times. During the night, there is almost no secretion at all. Purgative's (Carlsbad Salts) exercise no influence on its quantity, composition or power of digestion. 3rd. It contains no peptic (albumen digesting) ferment, and it is quite indifferent to all protean bodies. 4th. It changes starch into grape sugar. 5th. Cane sugar is transformed into grape sugar. 6th. Fats which contain free fatty acids, are emulsified by intestinal juice, but neutral fats are not."

---

Dr. L. Elsberg finds that by adding 50 per cent. saturated solution of potassi bichromate to a drop of blood, the red corpuscles change their form by indentation and protrusion. He also says, the red corpuscle has no separate investing membrane. "If it is really true that the red corpuscles are unattached bits of protoplasm, it is easy to understand how, after giving up their coloring matter, they may play an important part in the reparative process following extravasations."

---

The production of bromine from the brine pumped up from the salt pits in this country, is estimated at 1100 lbs. per diem.

It is now being exported to Europe. Its largest use is in the form of bromides. The French now use it in the production of a new aniline color.

---

There are yew trees in England "as old as the introduction of Christianity." It is said this tree attains to a greater age than any other European tree. "One at Hedson, in Bucks, surpassing all others in magnitude and antiquity." It was over 80 feet in circumference and was said to be 3,240 years old. "In all likelihood, the most ancient specimen of European vegetation."
—*Public School Journal.*

The electric light is now used for illuminating the reading room of the British museum. The new reading room contains 1,250,000 cubic feet of space, and with that of the surrounding libraries, reaches a total of 2,000,000 cubic feet. The book-worms can not complain of want of room for thought.

---

At the end of the year 1877, Europe had 268,800 miles of telegraphic lines; America 114,157 miles of wires; Asia (up to 1876) 24,521 miles of wires; Australia (1875) 23,582 miles of wires; Africa (1876) 8,148 miles of wires.

---

ONE grain of Sulphate of Eserine to one ounce of distilled water, and a drop or two in the eyes at night, is said to stimulate the function of accommodation, so that the use of spectacles may be delayed for several years.

---

THE Amalgamites will be pleased to learn that during the last three years, five counties of California produced over 6,448 tons of quicksilver.

---

VOLATILIZED petroleum is being successfully used in the manufacture of iron, and gives a better quality of product.

---

THE condition of national intelligence is so bad in Russia, that the native scientists are unable to publish their labors. Nihilism would do a good service if it produced an educational reform.

The French association for the advancement of science, has selected Algiers as the place for its next meeting in 1880.

---

Caves have been discovered near Stramberg, Moravia, which were inhabited by pre-historic man, as the bones of animals, and implements of bone and stone, together with articles of bronze and pottery, clearly prove.

---

At a musical festival held at Zurich, Switzerland, in the spring of 1878, five hundred of the guests were attacked by typhoid-fever, of whom one hundred died. It was found that they had partaken of the flesh of a sick calf, served to them by an inn-keeper. Let the M. D's tell how the disease originated.

---

The English now examine their seamen, to find the ability of the individual to distinguish between different colors Accidents at sea may sometimes be due to the inability of the lookout to distinguish colored lights.

---

The Electric light has been so sub-divided and distributed by a system of lenses and mirrors, that at a trial in San Francisco, two floors were illuminated from a single beam, without material loss of power.

---

Mr. Barwell, an English surgeon, recommends the use of a ligature made from the middle coat of the ox's aorta, for tying the large arteries. Its advantages are that, being smooth and flat, it does not cut the inner or serous coat; and the possibility of its becoming organized.

---

Consumption annually destroyes 3,000,000 of the earth's inhabitants. Dr. Talbot Jones says that a cold, dry, bracing climate, by increasing the demand for oxygen, is the best means of combating the disease.

---

By close investigation, Prof. Martin and Mr. Hartwell have come to the conclusion that the internal intercostal muscles are *expiratory*.

THE epiglottis has been removed from animals, and the function of deglutition continued unaffected.

